# The school environment and student health: a systematic review and meta-ethnography of qualitative research

**DOI:** 10.1186/1471-2458-13-798

**Published:** 2013-09-03

**Authors:** Farah Jamal, Adam Fletcher, Angela Harden, Helene Wells, James Thomas, Chris Bonell

**Affiliations:** 1 Institute for Health and Human Development, UH250, Stratford Campus, University of East London, Water Lane, London E15 4LZ, UK; 2 DECIPHer UKCRC Public Health Research Centre of Excellence, School of Social Sciences, Cardiff University, 1-3 Museum Place, Cardiff CF10 3BD, UK; 3 Barts Health NHS Trust, London, UK; 4 Department of Social and Environmental Health Research, London School of Hygiene and Tropical Medicine, 15-17 Tavistock Place, London WC1H 9SH, UK; 5 Department of Childhood, Families and Health, Institute of Education, University of London, 20 Bedford Way, London WC1H 0AL, UK; 6 Department of Social Policy and Intervention, Centre for Evidence Based Intervention, University of Oxford, Barnett House, 32 Wellington Square, Oxford OX1 2ER, UK

**Keywords:** Schools, Young people, Adolescent health, Health behaviours, Risk, Systematic review, Meta-ethnography, Qualitative

## Abstract

**Background:**

There is increasing interest in promoting young people’s health by modifying the school environment. However, existing research offers little guidance on how the school context enables or constrains students’ health behaviours, or how students’ backgrounds relate to these processes. For these reasons, this paper reports on a meta-ethnography of qualitative studies examining: through what processes does the school environment (social and physical) influence young people’s health?

**Methods:**

Systematic review of qualitative studies. Sixteen databases were searched, eliciting 62, 329 references which were screened, with included studies quality assessed, data extracted and synthesized using an adaptation of Noblit and Hare’s meta-ethnographic approach.

**Results:**

Nineteen qualitative studies were synthesised to explore processes through which school-level influences on young people’s health might occur. Four over-arching meta-themes emerged across studies focused on a range of different health issues. First, aggressive behaviour and substance use are often a strong source of status and bonding at schools where students feel educationally marginalised or unsafe. Second, health-risk behaviours are concentrated in unsupervised ‘hotspots’ at the school. Third, positive relationships with teachers appear to be critical in promoting student wellbeing and limiting risk behaviour; however, certain aspects of schools’ organisation and education policies constrain this, increasing the likelihood that students look for a sense of identity and social support via health-risk behaviours. Fourth, unhappiness at school can cause students to seek sources of ‘escape’, either by leaving school at lunchtime or for longer unauthorized spells or through substance use. These meta-themes resonate with Markham and Aveyard’s theory of human functioning and school organisation, and we draw on these qualitative data to refine and extend this theory, in particular conceptualising more fully the role of young people’s agency and student-led ‘systems’ in constituting school environments and generating health risks.

**Conclusion:**

Institutional features which may shape student health behaviours such as lack of safety, poor student-staff relationships and lack of student voice are amenable to interventions and should be the subject of future investigation. Future qualitative research should focus on health behaviours which are under-theorised in this context such as physical activity, sexual and mental health.

## Background

Childhood and youth are critical stages in the life-course for improving population-level health and reducing health inequalities. Multiple health-risk behaviours such as smoking, drinking, drug use (hereafter described collectively as ‘substance use’), violence and sexual risk are known to cluster together among the most disadvantaged groups of young people [1], suggesting the need for new common intervention strategies in schools [2]. This paper reports on a meta-ethnography of qualitative studies examining the processes by which schools’ social and physical environments influence young people’s health. This qualitative review was undertaken as part of a larger systematic review which also included theories and evidence from outcome and process evaluations and multi-level model (MLM) studies in order to build a comprehensive picture on how the school environment influences health [3]. Systematic reviews have consistently suggested that health education aiming to address these concerns by improving young people’s knowledge about health risks and modifying peer norms have relatively small and inconsistent results [4]. Socio-ecological approaches which address multiple-levels and contexts offer a complementary approach to changing behaviour via addressing upstream determinants [5]. These have the potential to ameliorate health inequalities [6]. One example of a socio-ecological approach is via interventions which change the school environment alongside curriculum-based education. This approach is supported by the World Health Organisation’s (WHO) framework for’Health Promoting Schools’ [7].

Markham and Aveyard [8] developed a theory of human functioning and school organisation, integrating theoretical conceptions of parenting [9] and cultural transmission in education [10]. Their theory focuses on how schools can promote health by enabling students to fulfil their capacity for autonomy, practical reasoning and affiliation through, what Bernstein termed, its ‘instructional’ and ‘regulatory’ orders. The instructional order is the way in which a school enables students to learn, both formally and informally. The regulatory order is the way in which a school aims to encourage norms of good behaviour and students’ sense of belonging. The theory suggests that schools in which many students become detached (from the regulatory order), disengaged (from the instructional order), and/or alienated (from both) will report poorer health outcomes. Schools can maximise student commitment to the instructional and regulatory orders by eroding unnecessary boundaries, for example between staff and students, and between different areas of learning; and by ensuring that both learning and decision-making in schools is student-centred.

Subsequent empirical research has aimed to test this theory. Three English studies [11-13] and one American study [14] found consistent evidence that schools with higher academic attainment and attendance than would be expected judging from the social profile of their students (which is an indirect measure termed ‘value-added’) had lower rates of substance use. For example, a longitudinal study by Tobler and colleagues [14] found that ‘value-added’ American high-school institutional environments have significantly lower rates of substance use and violence. These studies support a ‘school environment’ approach for reducing youth substance use and other risk behaviours [15]. However, these MLM studies of ‘school effects’ on student health only provide relatively weak evidence in support of a theory of human functioning and school organisation for several reasons. First, they rely on quite a crude measure of the school social environment based on a school-level summary score of the extent to which the students in the school achieved higher academic attainment and lower rates of truancy after accounting for their socio-demographic profile [16]. Second, the statistical correlations observed between higher value-added scores and lower rates of risk behaviours do not equate to direct evidence that students were more committed to the instructional and regulatory orders at these schools, nor what organisational factors influenced this. None of the MLM studies examined causal pathways.

Furthermore, these quantitative studies only offer very limited guidance on how the school context enables or constrains students’ health behaviours, or how students’ family backgrounds relate to these processes. For these reasons, qualitative evidence was included as part of the larger project to build a comprehensive picture on the effects of the school environment on young people’s health. Qualitative research is useful for exploring students’ lived experiences of schooling and how this may influence their health. This review reports the first meta-ethnography to address the question: *through what processes does the school environment (social and physical) influence student health outcomes?*

## Methods

The study adheres to PRISMA guidelines for systematic reviews. A PRISMA checklist is provided in an Additional file 1.

### Searching and evidence map

**Figure 1:**
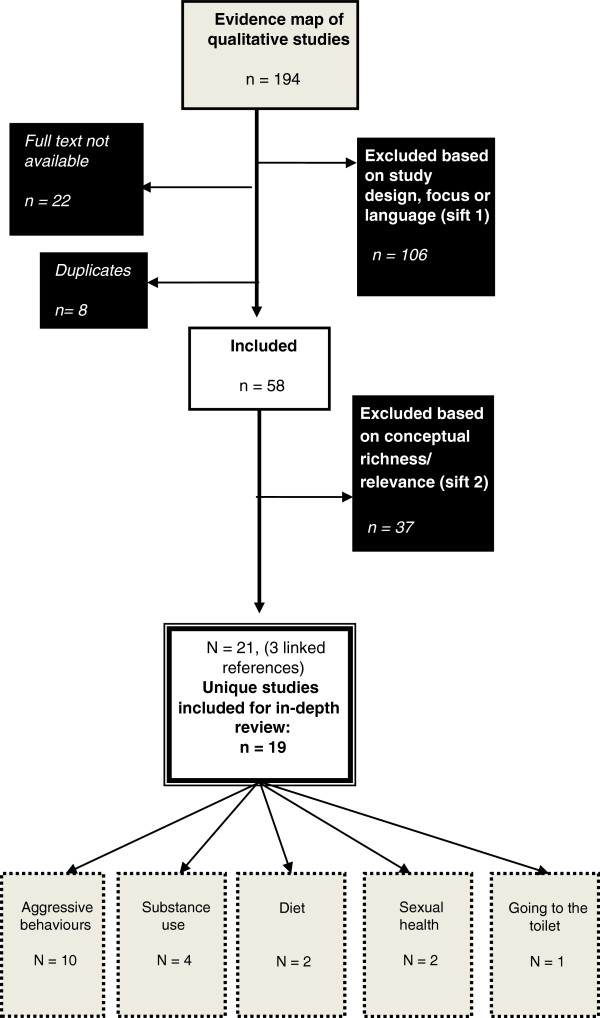
Flowchart of qualitative studies from evidence map to in-depth review.

The review was undertaken in two stages. In stage 1, sixteen bibliographic databases were searched between July and September 2010. A comprehensive approach to database searching was used in order to identify theory, outcome and process evaluations of school environment interventions, ecological and MLM studies of school effects as well as qualitative research on accounts of how school environment influences are implicated in health behaviours and outcomes (refer to Additional file 2). References (n = 82,775) were retrieved and screened to identify relevant studies (n = 1,144). Relevant studies were mapped (based on their titles and abstracts) to describe the types of question(s), setting(s) and population(s) they focused on. A diagram of the flow of literature through the review is provided in Figure 1 and the published protocol describes search strategies and exclusion criteria for stage 1 in detail [3]. An evidence map was produced and academic/policy stakeholders and young people were consulted to inform priorities for in-depth reviews (stage 2), which included the synthesis of qualitative research through meta-ethnography reported here. In-depth reviews focused on student (but not staff) health and were limited to studies which examine school environments in terms of: organisation and management; teaching, pastoral care and discipline; student attitudes and relationships with teachers; and physical environment.

### Exclusion criteria

Prior to the in-depth synthesis, references to qualitative research studies (n = 194) included in the evidence map were screened using the full text and excluded if they: were found to be not relevant on retrieval of the full paper; did not provide an account of how student health is influenced by features of the school environment; did not report on the aspects of school environment listed above; were not a qualitative study; or were not reported in English. Reports were double screened by two reviewers and any discrepancies were discussed until agreement was reached. A second set of criteria was then applied to all included reports in order to limit the review to relevant reports which provide findings conceptually rich enough to facilitate meta-ethnography. A scale of ‘high’, ‘medium’ and ‘low’ was used to rate: conceptual richness (i.e. do authors go beyond a description of the findings and interprets them to develop concepts, theories or metaphors?); relevance in terms of research aims; and relevance of findings for addressing our research question.

### Data extraction

We adopted an inclusive approach to data extraction [17] whereby reviewers extracted all relevant data presented in a study according to a standard proforma. Relevant data were: a) the study context (e.g. country, participant characteristics, sample size, research methods); and b) findings of the paper, highlighting themes or concepts which the study authors report and including author interpretation. Four reviewers extracted data, using the guidelines, on a randomly selected sample of two study reports to ensure thoroughness and consistency. All other reports were split between two reviewers and were checked by another reviewer and any disagreements were resolved by discussion. The data extracted provided a broad overview of the included studies, which is summarized in Additional file 3: Table S1. Reviewers however returned to reading full-text papers during the synthesis process in order to immerse themselves in the data. This is common in qualitative reviews where authors move between reading primary studies, data extraction, synthesis and interpretation in several cycles [17].

### Quality assessment

Studies that met the above criteria for inclusion were assessed for methodological quality using criteria from EPPI-Centre health promotion reviews [18]. The quality criteria addressed the rigour of: sampling; data collection; data analysis; the extent to which the study findings are grounded in the data; whether the study privileges the perspectives of children and young people; the breadth of findings; and depth of findings. The tool was piloted by four reviewers to ensure consistency and all remaining reports were assessed by two reviewers and checked by a third reviewer. Based on this assessment, reviewers rated the study overall on a ‘low’, ‘medium’ and ‘high’ scale. Reports were not excluded based on these quality assessment ratings; instead they were intended to inform our interpretation of findings.

### Synthesis

Studies were synthesized using a meta-ethnographic method adapted from Noblit and Hare’s [19] approach. This method involves treating interpretations and explanations in original studies as data and relating, translating and synthesising these ‘data’ sources via four steps.

Step 1: Reading and re-reading the studies to gain a detailed understanding of their findings, theories and concepts. To preserve the meaning of, and relationships between, concepts within an individual study, memos were used to describe ‘second order constructs’ (i.e. authors’ interpretation of the data) regarding how school-level influences on behaviour and health outcomes may occur.

**Figure 2:**
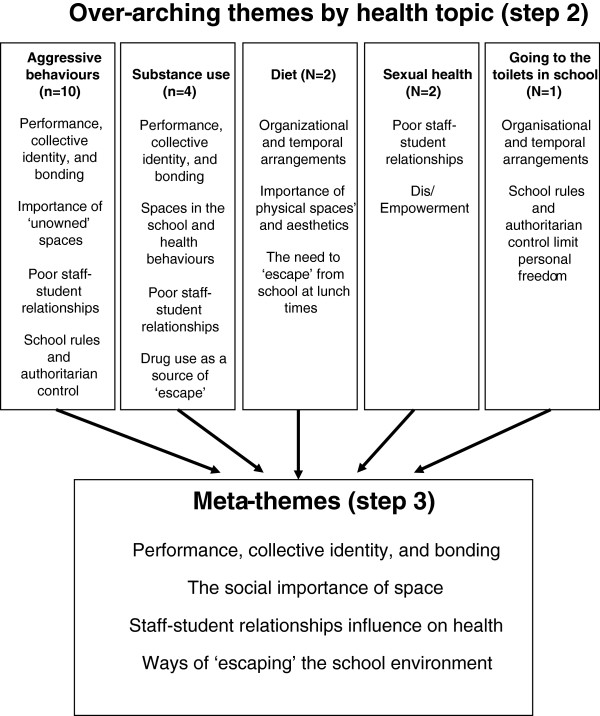
Reciprocal translation of included studies to develop meta-themes.

Step 2: In order to determine how the studies were related they were grouped according to health topics which the included studies were mostly concerned with (aggressive behaviours, substance use, diet, sexual health, and rules for going to the toilet) and the key concepts from individual studies within each health topic were synthesised, which resulted in lists of overarching themes for each of the five health topics (see ‘Figure 2’).

Step 3: Translating studies into one another to produce ‘meta-themes’ across the different health topics (see ‘Figure 2’). To draw out the findings under each meta-theme, studies rated ‘high’ in terms of their quality and/or conceptual richness were chosen as ‘index’ papers from which we extracted findings, and then compared and contrasted these findings with the findings of a second study, and the resulting synthesis of these two studies were then contrasted with a third study, and so forth. Noblit and Hare [19] refer to this as ‘reciprocal translation’.

Step 4: Synthesizing the (step 3) translation across health topics via interpretive reading of these meta-themes to develop a ‘line of argument’ regarding the process by which schools might influence health. This is presented in the discussion.

## Results

Nineteen studies were included in the meta-ethnography (summarised in Additional file 3: Table S1). Studies were conducted in the USA (n = 10), UK (n = 6), Australia (n = 1), South Africa (n = 1) and Sweden (n = 1). The majority of studies were conducted in high-school/secondary-school settings. A range of different socio-economic contexts and ethnic-minority groups were represented, although a disproportionate number of studies were conducted in disadvantaged urban contexts (n = 13) and none focused on rural settings. The results are presented below according to the four meta-themes based on the ‘reciprocal translations’ of studies (step 3).

### Performance, identity construction and bonding: acting ‘tough’

Several studies developed this concept and suggested young people often need to adopt ‘tough’ identities at school via acting aggressively and violently, and/or by engaging in substance use. Through such performances young people can foster close relationships with ‘tough’ peers and achieve ‘safety in numbers’. Students described as ‘geeky’ and who chose not to adopt ‘tough’ identities were vulnerable and isolated in disadvantaged, urban school contexts. This process of identity construction based on aggression and substance use thus appears to be an important source of bonding, social support and security, especially where young people feel educationally marginalised and/or unsafe [20-25].

“You smoke it [cannabis] for fun [but also] you wanna look bad. People think you’re a bad boy or bad girl… with me they are cool and I’m safe with the boys here” – female student, UK [25], p. 247.

One study explicitly developed the concept of violent incidents in schools as group performances through which the norms of acting ‘tough’ are collectively entrenched. This was evident in the way in which bystanders create a spectacle and space for violent behaviour:

“[They] were throwing punches at each other, trying to push each other’s head against the floor with all the strength that they could muster as they twisted their bodies together like twine. They were encircled by a ring of students locked arm-in-arm as they chanted in unison to the rhythm of the fighters” – ethnographic notes, USA [21], p. 51.

Through the diffusion of these norms, acting ‘tough’ often becomes entrenched in certain ‘high risk’, urban school environments [21,22,25]. This appears to reinforce existing patterns of health-risk behaviours, poor educational outcomes and teacher-student conflict in these schools, and both reflecting and exacerbating wider social and racial inequalities.

Reciprocal translation also led us to conclude that the norms around showcasing toughness may reflect the way in which the school environment maintains masculine conventions. Two studies found that young women were subjected to sexualized name calling (e.g. ‘slag’) and physical abuse (e.g. inappropriate touching) in schools [26,27]. This suggests that young men assert their power and reproduce existing gender inequalities in schools via such showcases of toughness.

### The social importance of space at school: health impacts

School spaces that are un-supervised appear to be ‘hotspots’ for certain health-risk behaviours. For example, aggressive behaviours and substance use were often associated with areas such as hallways, staircases, toilets, changing-rooms and empty classrooms [20,24,26,28]. Astor and colleagues [26] used the term ‘unowned’ to refer to these areas. In their study of five high schools, all 166 violent events reported by students could be mapped onto these ‘unowned’ spaces where few or no adults were present.

Several studies suggested that the large number of ‘unowned’ spaces in schools was the result of teachers focusing on classroom-based instruction and not the supervision of the wider school environment, which was considered beyond their professional responsibility [20,25,26,28]. Some school staff also reported avoiding potentially aggressive, ‘unowned’ spaces because of: fear of harm; the ambiguity of procedures; and inadequate support systems [26]. Where security guards, metal detectors and closed-circuit television cameras (CCTV) were used as alternative surveillance mechanisms in these ‘unowned’ spaces, students reported they were inappropriate and ineffective. For example:

“All the cameras are gonna do is videotape, you know what I’m saying? They’ll fight right in front of the camera too… some of them they’ll be asking, ‘Can I get that tape?” –male student, USA [26], p. 29.

Students reported that CCTV at best merely displaced risk behaviours to new ‘hotspots’ [25]. In some American high schools the deployment of security guards in such spaces was reported to facilitate new health-risk behaviours:

“Although the guards are discouraged by their superiors from ‘fraternizing’ with the students, they do often develop strong emotional relationships with them; we have known some guards who encourage students to study and to go to class; we have also known others who take drugs, sell drugs to students, have sex with them, and dispense favours” – ethnographic field notes, USA [20], p. 176.

Reciprocal translation also revealed connections between the spatial and social dynamics of school dining areas and student diet [24,29-31]. It appears that young people’s food choices are often constrained by the chaotic and unappealing aesthetic features of school dining areas [30,31]. For example, a study in Scotland described students’ frustrations at policies which organised lunch breaks by year-group and whether students want hot or cold food, which prevented them from eating lunch with friends and limited choice [30]. Aesthetically unappealing environments (e.g. no natural light, ‘cheap moulded chairs’, etc.) were also implicated in poor school meal uptake [31].

Another factor which seemed to influence lunchtime experiences was the presence (or non-presence) of teachers in dining halls. Multiple studies reported that teachers used lunch periods to prepare for afternoon lessons or have ‘breathing space’ away from students and that the lunch supervisors who ‘policed’ the dining halls did not make students feel safe, supported or comfortable, often eating quickly (if at all) to escape this environment [24,30].

### Teacher-student relationships influence on health

Studies consistently report that positive relationships between students and school staff, particularly teachers, are likely to be crucial to creating a healthy school environment [20,21,25,26,32-36] and that this may be particularly important for fostering students’ resiliency regarding substance use [37,38]. However, poor staff-student relationships were widely reported and this appeared to be a product of three inter-related features of the school environment.

First, young people consistently suggested that teachers were disconnected from the realities of their lives, especially urban Black youth [20,25,26] and students from the most disadvantaged and chaotic family backgrounds [27,34]. Teaching practices rarely engaged these young people, who then had fewer reasons not to engage in health-risk behaviours once disengaged from school:

“I think, if you’ve got no hope, if you’re surrounded by despair, then you don’t see that following the rules, that good work and good deed will get you anywhere” – teacher, USA [26], p. 26.

Furthermore, once students felt that staff did not understand them, this appeared to limit the extent to which staff could provide credible health messages and support them to make healthy transitions to adulthood – a theme which was reciprocated across studies of student diet and substance use [25,32]. Students also felt that ‘caring’ or ‘respectful’ teachers who defined their role beyond classroom based instruction were more effective in preventing and managing ‘risky’/‘problem’ behaviours [25,26,29].

Second, school rules to maintain discipline were usually said to be established without student input or consultation. This approach may be counter-productive as students recognize their lack of ‘voice’ and challenge the rules they feel are unfair and which disadvantage them [22,29,39,40], sometimes specifically through adopting health-risk behaviours, such as drug use [34]. Students also reported frustration at being treated as passive and child-like especially when already taking on adult-like responsibilities at home:

“I’ve had to be an adult for, like, my whole life really but oh no, they just think they always know best ‘cos they are the teacher and we are the students and we’ve gotta listen to them” – female student, UK [34], p. 555.

Third, teachers’ inconsistent application of rules was a recurring theme, which appeared to contribute to the poor student-staff relationships described above and also influence student health directly through a failure to prevent specific health-risk behaviours such as smoking and bullying on the school site [22,32].

Finally, the wider education system appeared partly to structure these poor institutional relationships and their adverse health consequences. In particular, high staff turnovers, a highly-divided market-orientated school system and target-based education policies focused on academic attainment were implicated in limiting the capacity for teachers to develop more supportive relationships [22,34].

“I can’t make anything happen here. I have no power… There’s nothing I can do. I have no voice” – teacher, USA [26], p. 25.

The market-orientated system whereby schools effectively compete for the ‘best’ students may also encourage teachers to keep problems such as aggression or drug use ‘hush-hush’ to maintain the reputation of the school, even if this meant that issues related to student health are never adequately addressed [22].

### ‘Escaping’ the school environment

Disengaged students often ‘escaped’ the school environment, which was implicated in their account of unhealthy habits. For example, students often reported that lunch-time provided a time of ‘relief’, to ‘hang out’ with friends and ‘escape’. Fast food was often eaten on the walk back to school or in local spaces surrounding the school that young people claimed as their own:

“Just usually run to try and beat all the queues for the food and then like we go down to the wee pigeon bit, sit, ate our lunch and then probably have a fag or two and then go back up the school” – student, UK [30], p. 462.

The need to escape the school environment at lunch periods had multiple implications for young people’s health: they were less likely to purchase healthy foods provided at school; more likely to visit local shops selling ‘junk’ food and high-calorie drinks; and more likely to smoke tobacco.

Using cannabis and other drugs was also reported as a potential means of escaping anxieties about school and as source self-medication in response to exam stress or a constant sense of academic failure [38]. A British female secondary-school student explained:

“If someone can’t be bothered about school, like you’re having a bad day then have a spliff in the morning and then it’s a good day. Pressure and stress can make people take drugs. If people don’t like the environment they’re in they are not going to be comfortable and getting on at school” – female student, UK [38], p. 131.

## Discussion

Our qualitative synthesis suggests complex pathways via which the school environment may shape health harms at a young age. Qualitative research forms a useful complement to quantitative studies on the health effects of the school environment. It illuminates how the school environment is understood by students from different backgrounds, and explores both students’ accounts of their actions and how these are enabled and constrained by the immediate school environment, and how wider structural forces such as education policies and students’ family backgrounds are implicated in this. Qualitative research can thus unpick how agency and structure are mutually constitutive and underlie social processes operating within schools which shape school effects on health.

Through an interpretation of the synthesis, below we present a ‘line of argument’ (step 4 in the meta-ethnography) about how schools might influence health. We refine Markham and Aveyard’s [8] theory of human functioning and school organisation to elaborate the importance of young people’s agency in constituting school structures, and the importance not merely of the instructional and regulatory orders of the school but also student social structures and networks. We argue that these two ‘systems’ are likely to interact in shaping school practices and influencing student health.

### Line of argument: the structuration of school organisation and student health

In line with Giddens’ [41] notion of structuration, two systems operate in the school environment: first, the student system (comprising peer-led processes and structures); and second, the school institutional system (comprising structures and processes involving school management, teachers, school staff and technologies such as CCTV). Students not only react to schools’ institutional systems for ordering instructional and regulatory practices, but they also promote their own parallel, competing versions of these instructional and regulatory ‘orders’ which Markham and Aveyard’s theory largely ignores. As well as their symbiotic relationship in shaping health, these systems are also both influenced by common social and structural factors beyond the boundaries of the school, such as students’ family backgrounds, which may constrain their sources of identity and social support, and education policies which constrain teachers’ time and responses.

We found that one of the most consistent and harmful effects of the student-led institutional system on health outcomes occurs via a process of normative social ‘instruction’ and the diffusion of highly-symbolic ‘regulatory’ styles based on practices such as intimidation, violence and drug use to (paradoxically) facilitate a sense of safety and security. Once these performative rituals permeate extended networks of students and become the norm, their social and symbolic importance reproduces the institutional ‘order’ through student-led social control, in extreme cases, in opposition to teachers and the schools institutional processes. Consider the rigid rules students reported following when confronted with a violent incident, such as linking arms around a ‘one-on-one-fight’: this collective performance helps establish bonding and collective identity.

Thus, *risk* arises from students developing the autonomy to engage in behaviour which is often regarded as anti-social but which is thoroughly social in its origins, rather than stemming from an absence of students’ practical reasoning, affiliation and autonomy as Markham and Aveyard suggest. This resonates with other ethnographically-driven theories explaining young people’s ‘street culture’ [42] and ‘tough fronts’ in inner city high schools [43], which conceptualise young people not merely as the victims of poverty and violence but as agents struggling for meaning and survival, and ultimately reinforcing existing educational, social and health inequalities.

‘Institutional authority’ [8] is also shaped by broader, cross-cutting socio-cultural structures which influence the process of localised, institutional structuration. For example, where students’ family and/or community culture is immersed in urban ‘street culture’, with relatively little hope of conventional social advancement, this will permeate the local student-network and thus shape both students’ actions and, in turn, the institutions’ regulatory response. State educational policies also provide an additional cross-cutting ‘structure’ that determine instructional and regulatory practices and, in turn, students’ health. For example, it appears that incentive structures such as ‘league tables’ in the UK and No Child Left Behind monitoring systems in the USA can create perverse incentives for schools to focus on more ‘academic’ students and neglect students’ general health and welfare. In the most extreme cases, the pressure of public exams or a constant sense of monitoring and surveillance can lead young people to seek sources of ‘escape’, either by engaging in substance use or by physically leaving school at lunchtime or for longer unauthorized spells.

### Limitations

We acknowledge that the way we have refined and extended Markham and Aveyard’s [8] theory is not without its problems. There is an apparent bias in the range and nature of qualitative research synthesised here. For example, the strong emphasis on a ‘disconnection’ between the top-down, school institutional regulatory and instructional ‘orders’ and the creative, student-led systems for social regulation and instruction could partly reflect the urban and disadvantaged context of the majority of the studies, where students and teachers may have the least in common. Nonetheless, the strength of the meta-ethnographic approach is that it combines evidence from multiple sources to increase validity and moves beyond merely providing a narrative review of individual studies and instead develops higher-order explanations. The value of this meta-ethnographic approach is also supported by the remarkable consistency in the findings of studies of variable quality undertaken in a wide range of settings, which differed by school system, deprivation level and ethnic make-up. However, some of these differences may have been masked in our review in the process of translating studies.

Another limitation is that we may have lost some of the meaning and depth of key concepts and themes during ‘step 2’ of the synthesis in order to translate themes across studies and identify meta-themes. However, we attempted to preserve individual authors’ interpretations by ensuring that all key concepts extracted from individual papers were accompanied by a narrative memo regarding how they were developed and connected in order to refer back to, and report, these relationships when synthesizing the findings across studies. Also, reports were not excluded based on ‘low quality’ scores as this could bias the review according to certain methodological approaches (e.g. interviews/focus groups rather than ethnographic approaches) and certain academic disciplines (e.g. anthropology) where methods may be less transparently reported. Studies, often from anthropology, that were rated as ‘low quality’ due to poor transparency in reporting of research procedure also provided the most conceptually rich data and thus contributed more substantively to the synthesis. Furthermore, the themes emerging in our review inevitably reflect the range of health topics covered in the primary qualitative studies. Most qualitative researchers exploring and theorising school level influences have focused mainly on how schools might shape risk behaviours, particularly aggressive behaviours and substance use and thus this review may be less useful for understanding how schools can support positive health and well-being, which should be the focus of future research.

The exclusion criteria were designed to identify those qualitative studies that were the most relevant to our review question and conceptually rich enough to facilitate a meta-ethnography approach which requires the presence and clarity of concepts for translation. Studies were excluded that did not examine how features of the school-environment (specifically, school type, physical environment, school management, teaching, support and discipline, student attitudes to school or relations with teachers) influences student health. We thus did not include a major body of work from sociology of education [44-46] including some studies that focused primarily on mental health. However, issues of self-esteem, anxiety and depression emerge prominently among the studies we’ve included in the context of substance use or aggressive behaviours for example, and this is in turn reflected in our synthesis.

### Implications for future research

There have been few conceptually rich qualitative studies focused on how the school environment as defined in this review might influence student diet and sexual health and none have passed our exclusion criteria that focus specifically on physical activity and mental health. While there is a body of research related to these topics, particularly from the field of sociology of education, further qualitative work oriented towards public health is needed. The bias in the literature towards young people in the most disadvantaged and extreme environments reflect the sociological research and theory more broadly and future studies should explore a range of contexts in order to include more ‘ordinary kids’ [45] who still represent the ‘missing middle’ [47]. The refined theory of human functioning and school organisation presented here should also be examined via quantitative and qualitative research in differing contexts (e.g. religious, rural/sub-urban, high SES and alternative schools).

The synthesis suggests how the school environment might be transformed to promote student health in future intervention studies. First, schools may promote student safety and health by ensuring teachers spend more time with students outside the classroom and by giving students more ‘voice’ regarding how schools are run. Second, interventions such as enhanced supervision and monitoring of school spaces that are ‘hot spots’ for student risk behaviour might be the focus for intervention. Third, policies could be developed to improve the social aspects of school food environments and to ensure students feel safe eating in school dining places where healthy eating is being promoted, for example by creating aesthetically appealing food environments where teachers eat with students, and where students have sufficient time and space to eat, as well as take a break with friends. The design of these programmes should be co-produced with students themselves so as to ensure they are appropriate and acceptable. However, such interventions should be examined in randomised controlled trials before being scaled up.

## Conclusion

In-depth qualitative studies suggest common pathways via which the school environment might shape young people’s health. Building on Markham and Aveyard’s [8] theory, our synthesis suggests that the student population not only reacts to the institutionally-directed instructional and regulatory ‘orders’, but is also an active agent in constituting its own instructional and regulatory structures. The separation of these two systems represents a lack of cooperative functioning, shared norms and understanding between students and the institutional ‘orders’; a condition most pervasive in urban contexts of disadvantage. In this context, students protect themselves and develop relationships by means of their own intervention: to build on Markham and Aveyard [8], the ways in which schools ‘order’ behaviour and learning indeed directly influences students’ reasoning, affiliation and ‘capacity’ for health but this is highly constrained, and not just by the organisation of the school, but also simultaneously by the organisation, norms and behaviours of the students themselves and their peers. The creative strategies students adopt also appear to produce a vicious circle whereby acting ‘tough’ or ‘escaping’ the school may lead to even more aggressive behaviours and higher rates of substance use, which in turn further reinforces and reproduces the boundaries between student-led and institutional social systems in new ways – an example of structuration in action.

## Competing interests

There are no conflicts of interests, including any financial, personal or other relationships with other people or organizations, which could inappropriately influence or be perceived to influence this work.

## Authors’ contributions

FJ conducted the review of qualitative research and led on the meta-ethnography synthesis. AF contributed to the design of the study and meta-ethnography synthesis. AH co-directed the project, contributed to planning the project and oversaw the meta-ethnography. HW contributed to screening and data extraction. JT advised on qualitative review methods, information management and commented on the report. CB conceived and directed the study, contributed to the meta-ethnography and commented on and contributed to the manuscript. The manuscript was drafted by FJ and AF. All authors read and approved the final manuscript.

## Pre-publication history

The pre-publication history for this paper can be accessed here:

http://www.biomedcentral.com/1471-2458/13/798/prepub

## Supplementary Material

Additional file 1 PRISMA 2009 Checklist. Click here for file

Additional file 2 Electronic databases searched. Click here for file

Additional file 3: Table S1 Included studies context, design and key themes. Click here for file
