# Supplementary material for: The school environment and student health: a systematic review and meta-ethnography of qualitative research
Source: BMC Public Health. 2013 Sep 3;13:798. doi: 10.1186/1471-2458-13-798 (PMC3844390; doi:10.1186/1471-2458-13-798)
Supplement: Additional file 2 — Electronic databases searched. [file 1471-2458-13-798-S2.doc]

Electronic databases searched

Sixteen bibliographic databases were searched between 30 July 2010 and 23 September 2010, with no limits on language or date:

- Australian Educational Index
- British Educational Index
- CAB Health (part of CAB Abstracts) – now known as Global Health
- The Campbell (C2) Library
- CINAHL (the Cumulative Index to Nursing and Allied Health Literature)
- Cochrane Controlled Trials Database
- Embase
- ERIC (Education Resources Information Center)
- HMIC (Health Management Information Consortium)
- IBSS (International Bibliography of the Social Sciences)
- Medline
- PsycInfo
- Social Policy and Practice (includes Child Data & Social Care Online)
- Social Science Citation Index (Web of Knowledge)
- Sociological Abstracts
- Dissertation Abstracts/Index to Theses.

Econlit and PAIS (Public Affairs Information Services) were also investigated but trial searches produced no new material.

Search terms

A broad approach to database searching was used in stage 1 given the cross-disciplinary nature of the review, the wide range of study designs to be included and the variability with which references were indexed in bibliographic databases. A sensitive search was undertaken using a large number of natural-language phrases. The search terms were used to develop *core searches* which comprised of the most relevant terms and where references were to be scanned carefully examining the full title/abstract in detail for inclusion; and *non-core searches* where a broader set of ‘non-core’ (or marginal) terms were applied and scanning for inclusion was to be done slightly more rapidly (although in practice both were scrutinized carefully). Some additional intervention terms were added to the key terms as a third searching phase.

*Core search*

- Setting (1) – school terms
- Population (2) – child terms
- Intervention/effect (3A) – key intervention/school-level effect terms
- Outcomes (4) – broad range of health outcomes.
- Key phrases (5)- related to health and schools

Search One: Set 1 and Set 2 and Set 3A and Set 4 (setting/population and key intervention/effects and outcomes).

Search Two: Set 5 (HPS phrases).

*Non-core search*

- Setting (1) – school terms
- Population (2) – child terms
- Intervention/effect (3B) – other non-key terms related to intervention/school-level effect (general free text)
- Outcomes (4) – broad range of health outcomes.
- Key phrases (5)- related to health and schools
- Key phrases (6) -Simple phrases combined with Set 4 outcome terms

Search Three: Set 6 and Set 4 (whole school phrases and outcomes).

Search Four: Set 1 and Set 2 and Set 3B and Set 4 (setting/population and key intervention/effects and outcomes). Additional terms were added to Set 3B in the third phase of the search.
