# Supplementary material for: The school environment and student health: a systematic review and meta-ethnography of qualitative research
Source: BMC Public Health. 2013 Sep 3;13:798. doi: 10.1186/1471-2458-13-798 (PMC3844390; doi:10.1186/1471-2458-13-798)
Supplement: Additional file 3: Table S1 — Included studies context, design and key themes. [file 1471-2458-13-798-S3.doc]

**Table 1: included studies context, design and key themes**

| **Study** | **Country** | **School type; grade(s)/age** | **Participants** | **Sample size (schools; participants)** | **Socio-economic profile/setting** | **Ethnicity** | **Research methods** | **Health topic** | **Key themes and concepts** |
| --- | --- | --- | --- | --- | --- | --- | --- | --- | --- |
| Astor et al. (1999) | USA | High school;  grades 9-12 | Students | 5; 78 | Socio-economic (SES) profiles: 2 schools had >85% students from low SES households; 1 school 60%; 1 school 23%; 1 school <10% | Ethnic/racial profiles: 2 school had >98% African American students; 1 had 50%; 2 schools had <15%. | Focus groups; interviews | Aggressive behaviour | (1) ‘Unowned’ spaces implicated in students’ of accounts of aggression  (2) Risk environment for gender violence  (3) Few ‘caring teachers’ / limited pastoral care  (4) The false promise / danger of new technologies (e.g. CCTV)  (5) Education policies ‘disconnected’ from the experiences of urban youth |
| Astor et al. (2001) | USA | Middle and Elementary schools; grades 2-8 | Students | 5; 377 | 82% of the students had free/reduced school meals | African American 51%; White 40%; Latino 5%; Other 4% | Mapping; interviews; school document analysis | Aggressive behaviour | (1) ‘Undefined spaces’ implicated in students’ accounts of aggression  (2) Lack of monitoring and supervision outside classroom  (3) Conflict in ‘overcrowded’ hallways and dining areas in middle schools |
| Brotman (2009) | USA | High school;  grades 7-12 | Students | 1; 20 | Specialist (urban) school training students in health professions | NR | Focus groups; interviews; observation | Sexual health | (1) The centrality of staff-student relationships to the school environment  (2) The value of empowerment |
| Burnet (1998) | South Africa | Secondary/ high school; NR | Students; staff | 1; 76 students; 3 staff | Rural township; ‘chronic poverty’ | “Coloured” township | Participant observation; interviews; group discussions;  questionnaires; diaries | Aggressive behaviour | (1) Violence as a functional tool for social control  (2) School hierarchy based on teachers authority and power |
| Brunson & Miller (2009) | USA | School type(s) high school; 13-19 | Young males ‘at-risk’ of / involved in crime | NR; 38 | Urban youth; high rates of poverty and crime; unemployment and lone-parent (female) families | African-American (racially segregated neighbourhoods) | Interviews | Aggressive behaviour | (1) Conflict and violence in schools a source of identity and status  (2) Spatial risk factors (e.g. ‘hot-spot’ areas which lack of teacher supervisions)  (3) Lack of confidence in new school surveillance technologies  (4) Schools- neighbourhoods overlapping contexts for gang-involvement |
| Cousins (1997) | USA | High school; NR | Students; school staff | NR | Low SES urban area | Mostly African-American | Participant observation/ ethnographic | Aggressive behaviour | (1) Symbolic violence  (2) Violence and aggression as a source of empowerment, identity and bonding  (3) Schools- neighbourhoods overlapping contexts for gang-involvement |
| Devine (1995) | USA | High school; NR | Students; school staff | NR | Low SES / high crime urban area | African-American | Participant observation/ ethnographic | Aggressive behaviour | (1) Students adopting ‘tough’ identities in violent school contexts  (2) Division of professional responsibility (i.e. guards = safety/security; teachers = cognitive learning)  (3) Poor teacher-student relationships in urban high schools |
| Fletcher et al. (2009a, 2009b, 2009c) | UK | Secondary schools; 14-15 | Students; teachers | 2; 30 students; 10 teachers | 2 London schools with varied SES profiles: 1 low free school meals (FSM) eligibility; 1 high FSM. | White-British 33%; Black 33%; dual heritage 23%; White-European 7%; other 4% | Interviews; observation; school document analysis | Substance use | (1) Drug use as a source of identity and bonding for disengaged students  (2) Diffusion of ‘safe’ identities (based on cannabis use and aggressive behaviour).  (3) New ‘counter school’ cultures explicitly based on students’ creative use of drugs, alcohol and tobacco  (4) Drug use and drinking as a source of escape from anxiety about school/exams  (5) School surveillance / CCTV harmful |
| Gordon & Turner (2001) | UK | Secondary School; 13-14 | Students; teachers | NR | Low SES area | NR | Focus groups; interviews | Substance use | (1) The ‘myth’ of students modelling teachers behaviour  (2) Positive teacher-student relationships and ‘respect’ promote school ethos |
| Haselwerdt & Lenhardt (2003) | USA | Middle and high schools; NR | Students | NR; 82 | 6 urban, suburban and rural school districts with varied SES profiles districts | NR | Focus groups | Aggressive behaviour | (1) Teacher-student disconnection; teachers lack of ‘respect’ (sub-theme)  (2) Lack of student voice in school policies & rules  (3) The limits of professional responsibility |
| Hosie (2007) | UK | Secondary school; NR | Students (all female) | NR; 93 | 10 ( urban and rural) school districts with varied SES profiles districts | >50% white | Interviews | Sexual health | (1) Dislike of school connected early pregnancy and other adverse outcomes  (2) The importance of ‘supportive’ teachers to the school environment  (3) School/education policies ‘unsupportive’ pregnant students |
| Keddie (2009) | Australia | High school; 14-15 | Students (girls only); teachers | 1 school: 4 students; 2 teachers | Working/middle-class area | White | Focus groups and interviews | Aggressive behaviour | (1) Teacher-student relationships characterised by masculine norms  (2) Masculine norms, power and entitlement in school  (3) Sexual harassment as a resource to gain power |
| Ludbland et al. (2010) | Sweden | Elementary/Middle schools; 9-16 | Students | 5; 19 | NR | NR | Interviews | Going to the toilets in schools | (1) School rules designed to ‘structure’ students’ health behaviours  (2) Teachers focus is on control of their classroom environment  (3) Lack of control and student anxiety over non-classroom environments |
| Marsiglia et al. (2002) | USA | Middle | Students (mostly young women) | NR; 60 | > 50% eligible for FSM | >50% Hispanic | Interviews | Substance use | (1) Attachment to school a ‘protective factor’  (2) Supportive teacher-student relationships foster ‘resiliency’ |
| Pike & Colquhoun (2009) | UK | Primary schools; 5-11 | Students; teachers; lunchtime supervisors | 2; NR | Low SES areas | NR | Interviews; focus groups; observations | Diet | (1) The social importance of the built environment / space  (2) Dining halls aesthetically unappealing  (3) Organisational and temporal arrangements of non-educational spaces not a priority for schools  (4) Separate spaces for staff and students to eat |
| Plano Clark et al. (2002) | UK | High schools; 16.3 (mean) | Students | 4; 205 | School setting: 2 urban; 1 sub-urban; 1 rural. | NR | Focus groups (students as co-researchers) | Substance use | (1) Spatial norms govern where health-risk behaviours (e.g. smoking) occur  (2) Rules enforced inconsistently and this leads to conflict with teacher |
| Waldron (2005) | USA | High | Students | 2; 31 | School 1: >50% middle class  School 2: approx. 50% low SES families | School 1: 83.6% white  School 2: 98.8% African American | Interviews; observation | Aggressive behaviour | (1) Implementation of school policies/rules is ‘messy’  (2) Differential treatment by social class/ ethnicity perceived as the norm  (3) Structural constraints on schools (e.g. staff turnover, education policies, etc.)  (4) Poor teacher-student relationships |
| Wills et al. 2005 | UK | Secondary school; 13-14 | Students | 1; 36 | Most deprived postcode areas | NR | Interviews | Diet | (1) The importance of organisational and temporal structures at lunchtime  (2) Students need to ‘escape’ the school grounds / gendered nature of ‘escape’ |
| Wilson-Simmons et al. 2006 | USA | Middle schools; grade 6-8 | Students; staff | 5; 54 students; 97 staff | Low SES urban areas | >90% African American | Focus groups | Aggressive behaviour | (1) Schools the site for violence because: young men need to adopt ‘tough’ identities; it’s a source of ‘entertainment’ and/or bonding  (2) Barriers to bystander intervention: poor student-teacher relationships; stigmatisation (“rat”, “squealer”, “snitch”, etc.) and fear of retribution |
